# Cultural malpractices during labor/delivery and associated factors among women who had at least one history of delivery in selected Zones of Amhara region, North West Ethiopia: community based cross-sectional study

**DOI:** 10.1186/s12884-021-03971-7

**Published:** 2021-07-12

**Authors:** Misganaw Fikrie Melesse, Yibelu Bazezew Bitewa, Kumneger Nigussie Dessie, Demeke Binalf Wondim, Tefera Marie Bereka

**Affiliations:** 1 Department of Midwifery, Debre Markos University, Debre Markos, Ethiopia; 2 Department of Psychology, Debre Markos University, Debre Markos, Ethiopia; 3 Department of Midwifery, Kotebe Metropolitan University, Addis Ababa, Ethiopia

**Keywords:** Cultural malpractice, Labor and delivery, Amhara region, Ethiopia

## Abstract

**Background:**

Every day, at least 810 women die worldwide from the complications of pregnancy and childbirth, 86% of which occurring in Southern Asia and Sub-Saharan Africa. One of the contributing factors for these problems is cultural malpractices during pregnancy and childbirth. The actual incidence of cultural malpractices in developing countries accounts for about 5–15% of maternal deaths. Thus, understanding the link between cultural affairs and maternal health is critical to saving the lives of women and their babies. Therefore, this research was aimed to assess cultural malpractices during labor and delivery and associated factors among women who had at least one history of delivery in selected Zones of the Amhara region, North West Ethiopia.

**Method:**

Community based cross-sectional study was conducted on women who had at least one delivery history in Awi, West, and East Gojjam Zones from January 1 to May 30, 2020. The multistage cluster sampling technique was used to select 845 study participants. Data was collected through a pre-tested and structured interview questionnaire, entered and cleaned using EPI info version 7.2, and exported to SPSS version 23 for analysis. Bivariable and multivariable logistic regression was employed to assess the association of the variables and a *P-*value less than 0.05 was declared as statistically significant.

**Result:**

Out of 845 women 162(19.2%) practiced nutritional taboo, 77(9.1%) women practiced abdominal massage and 273(32.3%) delivered their babies at home. Educational status of the respondents being un able to read and write (AOR = 14.35,95% CI: 3.12,65.96), husband's educational status (AOR = 3.80,95% CI: 1.24,11.64), residence (AOR = 2.93,95% CI: 1.41: 6.06), ethnicity (AOR = 2.20,95% CI:1.32, 3.67), pregnancy complications (AOR = 1.61,95% CI:1.02, 2.53), gravidity (AOR = 3.54,95% CI:1.38,9.08) and antenatal care follow up (AOR = 2.24, 95% CI:1.18,4.25) had statistically significant association with cultural malpractices during labor and delivery.

**Conclusion:**

This study showed that cultural malpractices during childbirth were high in Awi, West, and East Gojjam Zones relative to the country's maternal health service utilization plan. Working on antenatal care follow-up and women and husband education in a culturally acceptable manner may reduce cultural malpractices during labor and delivery.

## Background

Cultural malpractices are socially shared views and traditionally accepted behaviors experienced in a certain society that harm maternal health [1–3]. Worldwide, the period of labor and delivery is embedded with different beliefs, customs, and rituals in different societies that contribute a lot to maternal death [4]. Maternal mortality is unacceptably high worldwide accounting 295 000 deaths following pregnancy and childbirth. The vast majority of these deaths (94%) occurred in low-resource settings including Sub-Saharan Africa and Southern Asia accounted for approximately 86% of the estimated global maternal deaths. Sub-Saharan Africa alone accounted for roughly two-thirds (196 000) of maternal deaths, while Southern Asia accounted for nearly one-fifth (58 000) [5]. According to the Fragile States Index, Ethiopia is one of the fifteen countries which are considered as "a very high alert" for maternal death [6, 7].

Even though vigorous efforts are done by the state government of Ethiopia, national, and international non-governmental institutions to alleviate maternal death, it is still a serious problem accounting for 412 deaths per 100,000 live births [8]. In terms of service utilization, 74% of pregnant women had ANC follow-up but only 48% of women delivered at health institutional [9]. This indicated that many mothers suffer from complications of home delivery like infection of the reproductive tract and neonatal sepsis due to an unclean environment and inappropriate care during labor and delivery. Newborns improperly delivered by unskilled birth attendants, and the cultural malpractices performed during delivery are the major cause of sepsis and death. The actual incidence of maternal death due to cultural malpractices in a developing country is not known, but it accounts for 5–15% [10–12].

Cultural malpractices and beliefs greatly affect the healthcare-seeking behaviors of the women during childbirth [3, 13, 14]. Low utilization of institutional delivery rate, high maternal mortality, high infant and high cultural malpractices like home delivery are among manifestations of poor health care utilization in Ethiopia [15].

The majority of deaths and morbidity to women and their children can be prevented by appropriate utilization of reproductive, maternal, child, and neonatal health standards per the recommendation with the reductions of cultural malpractices [16]. In contrast, in areas where cultural malpractices are prevalent, they will adversely impact the health of the mother and her child [17].

Food taboos, abdominal massage, and home delivery are among cultural malpractices seen in Lao PDR as studied on ritual communities in 2015 [2].

Cultural malpractices are common in Ethiopia. For example, a study done in limmu genet, Ethiopia, indicated that nutritional taboo, abdominal massage, home delivery, and avoiding colostrum feeding to newborns were common findings [15].

Delaines on health care seeking behavior due to cultural malpractices at home lead to uterine rupture, severe bleeding, fetal distress, and finally fetomaternal death [4, 18].

After an effort of availing health facilities and enhancing access to health facilities, still, there is evidence of poor utilization of health care services in some parts of the countries in general and some regions in particular [19].

A little step was done in Ethiopia to increase institutional delivery by availing maternity waiting homes in the last months of pregnancy at the health facility with the fulfillment of certain ceremonies to reduce home deliveries [16]. However, some women delivered at home irrespective of maternity waiting for home availability.

Plenty of cultural malpractices directly or indirectly have an impact on the health of the mother and her baby like prelacteal feeding avoidance of colostrum and restriction of certain food types [20].

According to the 2019 Mini EDHS report, institutional delivery coverage was found to be only 48% but the ANC coverage was 74% [9]. This shows that the majority of women still delivered at home. So what? Women who had ANC follow-up should deliver at a health facility by principle since they had health care service utilization awareness.

As the nationwide gap in maternal and child health service utilization during childbirth necessitates research at the grassroots level.

Therefore, the main aim of this study was to assess cultural malpractices and associated factors among women who had at least one delivery history in Awi, East, and West Gojjam zones.

## Methods

### Study design

A community-based cross-sectional study design was used.

### Study area and period

This study was conducted in Awi, East, and West Gojjam zones (Debre Markos University research catchment areas) from February 1 to May 30, 2020.

**East Gojjam zone**: Debre Markos is its administrative center with a total population of 2,153,937 and 506,520 households who are distributed in 22 woredas and 480 Kebeles. There are 10 hospitals, 102 health centers, and 423 health posts in this Zone. **Awi zone**: Injibara is its administrative center and has a total population of 982,942 and 215,564 households. **West Gojjam zone**: West Gojjam zone: Finoteselam is its administrative center with total population of 2,106,596, of whom 1,058,272 men and 1,048,324 women [21].

### Source population

All women who had at least one history of delivery in Awi, East and West Gojjam Zones, Amhara Region, North West Ethiopia.

### Study population

All women who had at least one history of delivery in randomly selected Kebeles of Awi, East and West Gojjam Zones, Amhara Region, Northwest Ethiopia.

### Inclusion and exclusion criteria

#### Inclusion criteria

All women who had experienced at least one delivery and available during the data collection period were included in the study.

#### Exclusion criteria

Women who were severely ill that could not communicate verbally and those who were not lived at least for six months in the study area during the data collection period were excluded from the study.

### Sample size determination and sampling procedure

#### Sample size determination

The sample size was determined based on a single population proportion formula assumption. Using the prevalence of cultural malpractices from the study done in Meshenti town [17], west Gojjam, Amhara region, Ethiopia which is 50.9% with 5% confidence limit (margin of error) and 95% confidence interval.

$$\boldsymbol{n}={(\boldsymbol{Z}\frac{\boldsymbol{a}}{2})}^{2}\boldsymbol{*}\frac{\boldsymbol{p}(1-\boldsymbol{p})}{\boldsymbol{w}2}={1.96}^{2}\mathrm{*}\frac{0.509(1-0.509)}{({0.05)}^{2}}=384$$

where: *n* is the sample size.

*Zα*/2: with 95% confidence interval equal to 1.96.

*p*: estimation of cultural malpractice which is 50.9%

*w*: margin of error which is 1 − confidence level = 1–0.95 = 0.05.

Since it has two stages, we take a design effect of 2 and the sample size was 384*2 = 768. By considering a 10% non-response rate, the estimated number of non-response participants was 768*0.10 = 77.

Therefore, the minimum sample size for this study was 768 + 77 = 845.

### Sampling procedure

**Fig. 1:**
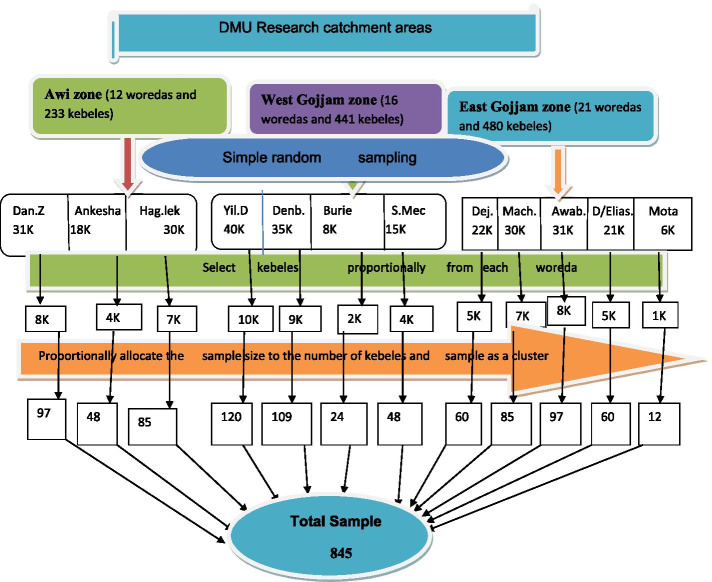
Schematic presentation of sampling procedure to assess cultural malpractices during labor and delivery and associated factors among women who had at least one history of delivery in selected Zones of the Amhara region, North West Ethiopia, 2020. Key: DMU: Debre Markos University, Dan.Z: Dangila zuria woreda, Fag.lek: Faguta Lekuma, Yil.D: Yilmana Densa, Denb: Denbecha, S.Mec: South Mecha, Dej: Dejen, Mach:Machakel woreda, Awab: Awabel woreda, D/Elias: Debre-Elias

A multistage cluster sampling technique was used. Twelve (12) Woredas were selected by using the lottery method from the three zones and cluster sampling was again employed after proportionally allocate the total sample size (845) to the selected kebeles of each woreda (Fig. 1).

### Study variables

#### Dependent

Cultural malpractices during labor and delivery.

#### Independent

Age, Education status, Marital status, Residence, Religion, Ethnicity, Income, Occupation.

History of abortion, ANC follow-up, Heath care-seeking, decision-maker, caregiver for home delivery.

### Operational definitions

**Labor**: a continuous process in which progressive uterine contraction results in the expulsion of the product of conception from the uterus through the birth canal after progressive effacement and dilatation of the cervix after viability of the fetus or after 28 weeks of gestation [22, 23].

**Delivery**: The release of the fetus and other products of conceptus from the uterus either vaginally or abdominally after viability of the fetus or after 28 weeks of gestation [22, 23].

**Cultural practices**: Experiences or beliefs that are socially shared views and behaviors practiced in a certain society at a certain time [2].

**Cultural malpractice**: Any cultural practice that can lead to an injurious, negligent, or improper practice which is accepted by a certain community [24].

**Home Delivery**: Child birth at home with culturally acceptable ceremonies by unskilled attendants [25]

**Food taboo**: Prohibitions of some foods for a certain occasions like pregnancy, labor/delivery or any others due to cultural and religious influences [26].

**Abdominal massage**: Hand-based downward rubbing of the pregnant woman's abdomen to shorten the labor duration and alleviating pain by using lubricants commonly butter [18].

### Data quality control

To assure the data quality, the data collection tool was pretested to check its clarity, and training was given for data collectors and supervisors regarding the objectives of the study, data collection method, and the significance of the study. Daily communication was conducted among data collectors, supervisors, and principal investigators for discussion regarding presenting difficulties and to assess the progress of data collection. Collected data was checked for completeness and on spot corrective measures were taken by data collectors and supervisors.

### Data processing and analysis

All collected questionnaires were rechecked for completeness and coded. Then these data were entered and cleaned using Epi Info 7.2 software and exported to SPSS version 23 for analysis. Bivariable logistic regression was employed to identify an association, and a multivariable logistic regression model was used to control the effect of confounders.

Variables having *P-*value less than 0.05 in the Bivariable analysis were fitted into the multivariable logistic regression model. Ninety-five percent confidence interval of odds ratio was computed and a variable having *P-*value less than 0.05 in the multivariable logistic regression analysis was considered to declare statistical significance.

Before the actual logistic regression analysis was done, the necessary assumption of the logistic regression model was checked by using the Hosmer–Lemeshow test of goodness of fit which has a chi-square distribution.

For further analysis, descriptive statistics like frequencies and cross-tabulation were performed. Graphical presentations such as bar charts and pie charts were used to present the result findings of the study in addition to texts and tables.

## Results

### Socio-demographic characteristics

A total of 845 respondents were included in this study with a 100% response rate. The mean age of the respondents was 35.2 years (SD ± 8.9) ranging from 18 to 75 years as minimum and maximum ages respectively. Among the participants, five hundred forty-five (64.5%) were rural dwellers. According to the web page of the Ethiopian living wage series per month, more than one-third, 284 (33.6%) of the study participants responded that their monthly family income was < 2037 Ethiopian birr [27] (Table 1).

**Table 1:** Socio-demographic characteristics to assess cultural malpractices during labor and delivery and associated factors among women who had at least one history of delivery in selected Zones of the Amhara region, North West Ethiopia, 2020 (*n* = 845)

Variable	Frequency	Percent
**Age (**in years**)**		
≤ 20	8	0.9
21 -35	491	58.2
≥ 36	346	40.9
**Marital status**		
Single	10	1.2
Married	698	82.6
Widowed	87	10.3
Divorced	50	5.9
**Religion**		
Orthodox	794	94.0
Muslim	45	5.3
Protestant	4	0.5
Catholic	2	0.2
**Educational status**		
Unable to read and write	339	40.1
Read and write	227	26.9
Primary education	106	12.5
Secondary education	73	8.7
College and above	100	11.8
**Ethnicity**		
Amhara	709	83.9
Agew	136	16.1
**Occupation**		
Housewife	341	40.4
Governmental worker	74	8.8
Merchant	69	8.2
Farmer	286	33.8
Student	7	0.8
NGO	3	0.4
Private worker	52	6.2
Others	13	1.5
**Husband’s Educational status**		
Unable to read and write	160	22.9
Read and write	250	35.7
Primary school	97	13.9
Secondary school	71	10.1
College and above	122	17.4
**Residence**		
Rural	546	64.6
Urban	299	35.4
**Income**		
Income < 2037	284	33.6
Income from 2037 to 3506	277	32.8
Income > 3506	284	33.6

### Obstetrics characteristics

These factors are directly related to pregnancy and services towards pregnancy including ANC follow-up, abortion management, delivery care, and the care given for the newborn.

These services may be influenced by many factors like cultures, beliefs, and rituals in addition to other factors. More than three fourth (75.4%) of the respondents had ANC follow-up in the study area. Five hundred eighty-two (68.9%) respondents were a decision-maker by themselves to get maternal health care services (Table 2).

**Table 2:** Obstetrical characteristics to assess cultural malpractices during labor and delivery and associated factors among women who had at least one history of childbirth in selected Zones of the Amhara region, North West Ethiopia, 2020(n = 845)

Variables	Frequency	Percent
**History of abortion**		
No	640	75.74
Yes	205	24.26
**ANC follow up**		
No	208	24.6
Yes	637	75.4
**Reason for no ANC**		
No health facility nearby	25	12.0
I don't understand it's function	133	63.9
Unwillingness of family	38	18.3
Others	12	5.8
**Decision maker for health care**		
My self	582	68.9
Husband	226	26.7
Husband’s mother	37	4.4
**Who attend the delivery at home**		
Family	204	77.3
TBA	57	21.6
Neighbors	3	1.1

**Fig. 2:**
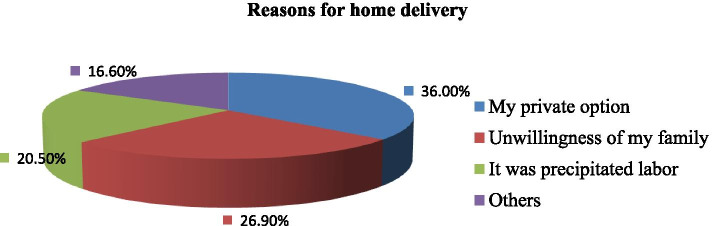
Reasons for home delivery among women who had at least one history of childbirth in selected Zones of the Amhara region, North West Ethiopia (n = 264). Others: From the above figure represent transport cost due to COVID-19 and fear of the infection itself

Two hundred sixty-four (31.2%) of the respondents were delivered at home for different reasons. More than one-third of the participants considered their private options as a reason for home delivery (Fig. 2).

**Fig. 3:**
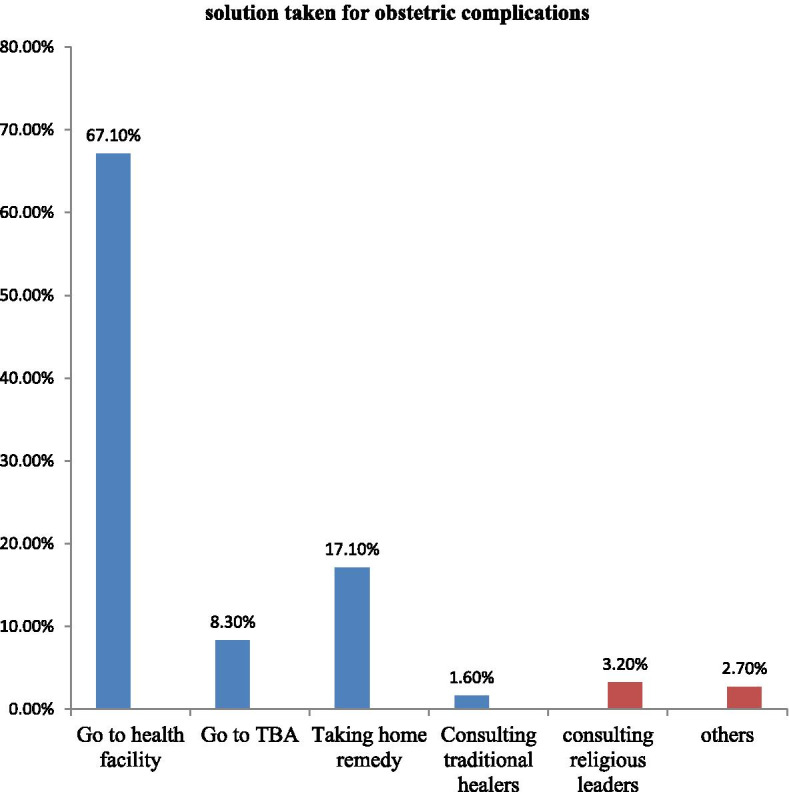
Solutions taken for past obstetric complications among women who have one history of childbirth in Awi, East and West Gojjam Zones, Amhara Region, North West Ethiopia, 2020. Represents relieves by itself without taking anything, preparing red Jano (gabi with the red stripe at the end) and rotating it around the women’s head

Among the respondents two hundred fifty-two (29.8%) were faced past obstetric complications and take different solutions for those problems like going to the health facility, taking home remedies, and others in general according to their cultures and beliefs (Fig. 3).

### Cultural malpractices

Cultural malpractices contribute a lot for fetomaternal morbidity and mortality during labor and delivery in different ways. For example, by potentiating the three delays and making them negligent for medical care. Therefore, this study assessed the respondents’ experiences of malpractices during labor and delivery. Out of 845 women 162 (19.2%) practiced nutritional taboo, 77(9.1%) women practiced abdominal massage and 264 (31.2%) delivered their babies at home (Table 3).

**Table 3:** Prevalence of cultural malpractices during labor and delivery and associated factors among women who had at least one history of childbirth in selected Zones of the Amhara region, North West Ethiopia, 2020

Variables	Frequency	Percent	95% CI	
			Lower	Upper
**Place of delivery**				
Health facility	581	68.8	65.6	71.7
Home	264	31.2	28.3	34.4
**Food taboo during L&D**				
Yes	162	19.2	16.3	22.1
No	683	80.8	77.9	83.7
**Abdominal massage during L&D**				
Yes	77	9.1	7.3	11.0
No	768	90.9	89.0	92.7

### Factors associated with cultural malpractices

Nutritional taboo was cross-tabulated against sociodemographic and obstetrical characteristics. Bivariable and multivariable analysis was conducted to assess if there was a significant association between them. There was a significant association between residence, ethnicity, gravidity, and pregnancy-related complications of respondents, and nutritional taboo (Table 4).

**Table 4:** Association of nutritional taboo with socio-demographic and obstetrical factors among women who had at least one history of childbirth in selected Zones of the Amhara region, North West Ethiopia, 2020

Variables	Food taboo		*P-*value	COR(95%:CI)	*P-*value	AOR(95%:CI)
	No	Yes				
**Residence**						
Urban	285	14		1		1
Rural	398	148	0.0001	7.57(4.29:13.37)	**0.004**	2.93(1.41: 6.06)^*^
**Ethnicity**						
Amhara	607	102	1			1
Agew	76	60	0.0001	4.70(3.16: 6.99)	**0.002**	2.20(1.32: 3.67)^*^
**Pregnancy complications**						
No	501	92	0.0001	2.09(1.47: 2.99)	**0.040**	1.61(1.02: 2.53)^*^
Yes	182	70		1		
**Decision maker**						
Myself	486	96		1		1
My husband	169	57	0.005	1.71(1.18: 2.48)	0.419	1.20(0.77: 1.88)
My husband’s mother	28	9	0.223	1.63(0.74: 3.56)	0.211	1.98(0.68: 5.74)
**Gravidity**						
Gravida = 1	148	7		1		1
Gravida 2—5	433	89	0.0001	4.35(1.97: 9.59)	0.079	2.16(0.92: 5.12)
Gravida ≥ 6	683	162	0.0001	13.68(6.03:31.03)	**0.009**	3.54(1.38: 9.08)^*^

**Key**: 1 = Reference, * = *p-*value less than 0.05

Home delivery was cross-tabulated against sociodemographic and obstetrical characteristics. Bivariable and multivariable analysis was conducted to assess if there was a significant association between them. There was a significant association between educational status, husband's education, and ANC follow-up of respondents and place of delivery (Table 5).

**Table 5:** Association of home delivery with socio-demographic and obstetrical factors among women who had at least one history of childbirth in selected Zones of the Amhara region, North West Ethiopia, 2020

Variables	Home delivery		*P-*value	COR(95%: CI)	*P-*value	AOR(95%: CI)
	**No**	**Yes**				
**Residence**						
Urban	258	41		1		1
Rural	324	222	0.000	4.31(2.98:6.25)	0.435	0.69(0. 28: 1.74)
**Educational status**						
Unable to read and write	167	172	0.0001	16.14(6.88:37.84)	**0.0001**	14.35(3.12: 65.96)^**^
Read and write	160	67	0.0001	6.56(2.74:15.71)	**0.005**	9.04(1.97: 41.52)^*^
Primary school	91	15	0.060	2.58(0.96:6.95)	0.140	3.34(0.67: 16.62)
Secondary school	70	3	0.582	0.67(0.16:2.78)	0.870	1.19(0.16: 8.99)
College and above	94	6		1		1
**Husband educational**						
Unable to read and write	100	60	0.0001	14.04(5.43:36.32)	**0.019**	3.80(1.24: 11.64)^*^
Read and write	149	101	0.0001	15.86(6.26:40.20)	**0.0001**	7.69(2.69: 22.01)^**^
Primary school	79	18	0.001	5.33(1.90:14.95)	**0.045**	**3.36(1.03: 10.97)**
Secondary school	66	5	0.379	1.77(0.50:6.35)	0.967	0.97(0.21: 4.42)
College and above	117	5		1		1
**ANC follow up**						
Yes	550	77		1		1
No	32	186	0.0001	41.52(26.62:64.76)	**0.0001**	34.94(19.74:61.86)^**^
**Gravidity**						
Gravida = 1	134	21		1		1
Gravida 2—5	379	143	.001	2.41(1.46:3.96)	0.510	1.32(0.58: 3.02)
Gravida ≥ 6	69	99	.000	9.16(5.27:15.92)	0.068	2.47(0.93: 6.52)

**Key**: 1 = Reference, ** = *P-*value less than 0.001, * = * p-*value less than 0.05

Cross-tabulation was done for abdominal massage during labor and delivery against sociodemographic and obstetrical characteristics when data analysis. ANC follow-up was the only variable that was significantly associated with the abdominal massage of respondents by both bivariable and multivariable analyses (Table 6).

**Table 6:** Association between abdominal massage and socio-demographic and obstetrical factors among women who had at least one history of childbirth in selected Zones of the Amhara region, North West Ethiopia, 2020

Variables	Abdominal massage		*p-*value	COR(95%:CI)	*P-*value	AOR(95%:CI)
	No	Yes				
**Gravidity**						
Gravida = 1	150	5		1		1
Gravida 2—5	487	35	0.115	2.16(0.83: 5.60)	0.388	1.74(0.49: 6.13)
Gravida ≥ 6	131	37	0.0001	8.47(3.24:22.19)	.074	3.44(0.89: 13.34)
**ANC FOLLOW UP**						
No	173	45	0.0001	4.84(2.98: 7.85)	**0.013**	2.24(1.18: 4.25)^*^
Yes	595	32		1		1
**Pregnancy complication**						
No	558	35	0.004	0.31(0.17:0.62)	0.267	1.47(0.74: 2.92)
Yes	210	42		1		1
**History of abortion**						
No	600	40	0.0001	3.30(2.05: 5.33)	0.208	1.58(0.78: 3.21)
Yes	168	37		1		1
**Husband education**						
Unable to read and write	138	22	0.010	3.73(1.37:10.16)	0.397	1.60(0.54: 4.79)
Read and write	232	18	0.250	1.81(0.66: 5.01)	0.849	1.11(0.38: 3.25)
Primary school	95	2	0.404	0.493(0.09:2.60)	0.308	0.42(0.08: 2.24)
Secondary school	67	4	0.627	1.40(0.36: 5.40)	0.658	1.36(0.35: 5.40)
College and above	117	5		1		1

Key: 1 = Reference, * = * p-*value less than 0.05

## Discussion

This community-based cross-sectional study was conducted to assess cultural malpractices and associated factors among women who had at least one delivery history in selected zones of Amhara region, North West Ethiopia, 2020.

The result of this study showed that the magnitude of home delivery was 31.2% with 95% CI: 28.3 to 34.4 (Table 3). This finding is in line with the studies done in Meshenti town (29.7%) [17] and Serra Leon (31.1%) [13]. This similarity can be explained by the methodology, sample, and size we used. As the studies in Meshenti town and Serra Leon, we use a cross-sectional study design with enough sample size. However, our result is lower than the study conducted in South West Ethiopia (38.3%) [15]. This may be due to a great difference in culture, civilization, and ethnicity between South West and North West Ethiopian people. In southern Ethiopia, there are a lot of ethnic groups with different culture and most leads nomadic lifestyle whereas, in North West part, Amhara ethnic group is dominant with an almost similar culture and farming lifestyle.

The other possible explanation for this difference could be the period in which the study was conducted. As time has gone, the awareness of the community about the health service utilization will increase and again accessibility of those health services will replace cultural practices and traditional beliefs concerning fetomaternal health during labor and delivery.

This finding is also lower than the results from the 2019 Mini-EDHS report which is 52% [9] and the study done at the country level, Ethiopia (67.2%) [28]. The difference might be due to the study area. The EDHS report and country-level study were nationwide findings while our finding was done in three zones of one region. The other possible reason for this difference could be the sampled population size and cultural diversity throughout the country.

Our finding is again much lower than the findings of the studies done in rural Nepal (87%) [4] and Kenya (66.7)%) [29] The possible explanation for this difference may be the study setup. The studies in Nepal and Kenya were conducted in rural areas while our study was done both in rural and urban. People who live in urban areas have more health service access than rural ones. Again there may socio-cultural differences between Ethiopia and these countries.

Maternal educational status is one of the factors associated with home delivery by multivariable analysis (Table 5). Women who were unable to read and write were 14.35 times more likely to deliver at home when compared with women whose educational status was college and above (AOR = 14.35,95% CI:3.12: 65.96). Women whose educational status were read and write were 9.04 times more likely to deliver at home as compared to their counterparts (AOR = 9.04,95% CI: 1.97: 41.52).

This finding is in agreement with studies conducted in Southern Ethiopia [15], Eritrea [30], Nepal [25], India [3], and Turkey [31].

The possible explanation could be, respondents who were unable to read and write had a low level of understanding about health care service utilization and healthcare-seeking behavior rather they want traditional healers and cultural home remedies, on the contrary, those women who were educated had better information about health and have information where they can get health care during labor and delivery.

This also indicates that as women are educated, they might minimize the influence of cultural practices during labor and delivery that helps them to prefer institutional delivery over home delivery.

Husband's educational status was the other factor that was significantly associated with home delivery (Table 5). Women whose Husband's educational status were unable to read and write were 3.8 times more likely to deliver at home when compared with women whose Husband's educational status were college and above (AOR = 3.80,95% CI:1.24: 11.64). This finding is supported by a study conducted in Eritrea [30]. This is possibly explained since in most communities' husbands are the decision-maker concerning health care. When the husbands are educated and know about the risk of home delivery, they allow their wives to deliver at the health institution.

After adjusting the potential confounders, ANC follow-up was a factor significantly associated with home delivery (Table 5). Respondents who had no ANC follow-up were 34.94 times more likely to deliver at home as compared with women who had ANC follow-up (AOR = 34.94,95% CI: 19.74:61.86). This result is in concurred with studies conducted in Gambella Region, Ethiopia [32], South Sudan [33], Zambia [34] and Malaysia [35]. The possible justification for this could be women who have visited a health facility for ANC service, will have awareness of the risks and complications of home delivery. Again when they have ANC follow-up, they get the opportunity to be familiar with the health professionals and know the reality of what is done in the health facility by avoiding cultural rumors.

The other possible explanation might be when pregnant women had ANC follow-up, they will get a waiting room in the last month of pregnancy especially for those who are far apart from the health institution so that they utilize the delivery service effectively by breaking the influence of cultural practices and traditional beliefs during labor and delivery.

**Nutritional taboo was** the other cultural malpractice which was assessed in this study. The prevalence of nutritional taboo during child birth in selected zones of Amhara region was found to be 19.2 with 95% CI: 16.3–22.1 (Table 3). This result is in line with the studies done in Meshenti town (19.5%) [17] and Limmu Genet town, Ethiopia (19.1%) [36]. The possible explanation for this similarity might be the socio-cultural similarities between Meshenti town and our study setting. However, the result of this study was lower than the finding of the study done in Awabel district, Ethiopia (27%) [37]. This difference might be due to the study area and the sample size we use. This is because we have studied on the three zones with relatively large sample size than the study done on one district. Again our finding was higher than the study done in the Tigray region (12%) [38]. This difference may be possibly explained by awareness and socio-cultural difference. People in the Tigray region are more educated, civilized and near for information than people in the Amhara region.

Residence, ethnicity, gravidity and pregnancy complications were the factors that significantly associated with food taboo during labor and delivery (Table 4). Respondents who were rural dwellers were 2.93 times more likely to practice nutritional taboo when compared with their counterparts (AOR = 2.93,95% CI: 1.41: 6.06). This finding is supported by studies done in southern Tigray [39], Arsi, Central Ethiopia [20] and Sera Leon [13]. The possible explanation could be women who live in rural area are far from the information about the risk of food prohibition (maternal dehydration that leads to exhaustion, fetal distress and death) during labor and delivery than women who are urban dwellers.

Respondents who were Agew in their ethnicity were 2.20 times more likely to practice nutritional taboo than Amhara ethnic groups (AOR = 2.20, 95% CI: 1.32: 3.67). This finding is in agreement with the studies done in Lao PDR [40] and India [3]. The possible reason could be the socio-cultural differences of the two ethnic groups.

Women with high order of pregnancy (gravida ≥ 6) were 3.54 times more likely to practice nutritional taboo than women with low order pregnancy (AOR = 3.54, 95% CI: 1.38: 9.08). This finding is supported by studies held in Debre tabor, south Gondar, Ethiopia [41] and Southeast Ethiopia [42]. The reason might be women with gravida 6 and above mostly aged, illiterate and culture-dependent than gravida one women. Thus, it is so difficult to change their attitude towards cultural malpractices during childbirth including nutritional taboo.

Respondents who had developed pregnancy related complications were 1.61 times less likely to practice nutritional taboo than their counterparts (AOR = 1.61, 95% CI: 1.02: 2.53). This finding is similar to the study done in South Africa [12]. The possible justification for this finding could be women who had exposed for pregnancy complications may get the opportunity to have health education about the importance of taking liquid foods while they were on labor from the health care providers during the management of their complication and they may learn from the complication they face than respondents who had no any pregnancy complications.

**Abdominal massage** during labor and delivery was another cultural malpractice which we emphasized in this study. The prevalence of abdominal massage during childbirth in our study setup was found to be 9.1 with 95% CI: 7.3–11.0 (Table 3) which is lower than the studies done in Meshenti town (24.5%) [17] and Southwest Ethiopia (22%) [15]. This could be possibly explained by the time and the sample size of the study. The study in Meshenti was done in 2016 and the south west Ethiopia was conducted in 2015 while our study was done in 2020. This indicates there is at least a 5 years’ gap, so that the community awareness about cultural malpractices effect on pregnancy outcome recently is better than the 5 years ago. This may make our result lower than the previous studies.

After adjusting the potential confounders, only ANC follow-up was significantly associated with abdominal massage during labor and delivery (Table 6). Women who had ANC follow-up were 2.24 times less likely to practice abdominal massage during childbirth than women who had no ANC follow up (AOR = 2,24, 95% CI: 1.18: 4.25). This finding is supported by a study done in Nigeria [43]. The reason for this finding might be when women have ANC follow-up they can get the opportunity to be counseled about complications of cultural malpractices than women who have no ANC follow-up. Again women with ANC follow-up mostly delivered at the health institutions by skilled health professionals than their counterparts. Thus, women who delivered at health facility may have no any chance to practice abdominal massage during childbirth culturally.

### Limitation

Difficulty of data collection due to Covid-19 in terms of cost and getting full information from the respondents.

## Conclusion

This study revealed that cultural malpractices during labor and delivery was high in Awi, West, and East Gojjam Zones relative to the country’s maternal health service utilization plan.

Educational status, husband’s educational status, gravidity, pregnancy-related complications, residence, ethnicity and ANC follow-up had a statistically significant association with cultural malpractices during labor and delivery.

## Data Availability

The data sets used and analyzed during the current study will be available from the corresponding author on reasonable request.

## Abbreviations

ANC: Antenatal care
AOR: Adjusted odds ratio
CI: Confidence interval
CMP: Cultural malpractice
FMOH: Federal Ministry of Health
HEW: Health extension worker
HD: Home delivery
LD: Labor delivery

## References

[1] Frese M. Cultural practices, norms, and values. J Cross-Cultural Psychol. 2015;46.

[2] Sychareun V, Phengsavanh A, Hansana V, Somphet V, Menorah S (2009). Cultural beliefs and traditional rituals about child birth practices in Lao PDR.

[3] Young Oak, Wells a ED. Childbearing traditions of Indian women at home and abroad: An integrative literature review. Austral College Midwives. 2014. 10.1016/j.wombi.2014.08.006 25257377

[4] Chand SB, Cultural beliefs and traditional rituals about child birth practice in rural, Nepal. MOJ Public Health 2016, 5(1).

[5] WHO U, UNFPA, World Bank Group and the United Nations Population Division (2019). Trends in maternal mortality: 2000 to 2017.

[6] Ganchimeg TOE, Morisaki N (2014). Pregnancy and childbirth outcomes among adolescent mothers: a World Health Organization multicountry study. BJOG..

[7] Althabe FMJ, Gibbons L (2015). Adverse maternal and perinatal outcomes in adolescent pregnancies: the Global Network’s Maternal Newborn Health Registry study. Reprod Health..

[8] ICF. CSACEa: Ethiopia Demographic and Health Survey 2016: Key Indicators Report. Ethiopia; 2016.

[9] MOH. Mini Demographic and Health Survey. Addis Ababa: Ethiopian Public Health Institute; 2019.

[10] Central statistical authority: Ethiopian demographic & health survey . Addis Ababa; 2011.

[11] Lewis G (2014). The cultural environment behind successful maternal death and morbidity reviews. BJOG Int J Obstetr Gynaecol.

[12] Marabele PM, Maputle MS, Ramathuba DU, Netshikweta L (2020). Cultural factors contributing to maternal mortality rate in rural villages of Limpopo Province South Africa. Int J Womens Health.

[13] Sharkey A, Yansaneh A, Bangura PS, Kabano A, Brady E, Yumkella F, Diaz T (2016). Maternal and newborn care practices in Sierra Leone: a mixed methods study of four underserved districts. Health Policy Planning.

[14] Turner C, Pol S, Suon K, Neou L, Day NPJ, Parker M, Kingori P (2017). Beliefs and practices during pregnancy, post-partum and in the first days of an infant’s life in rural Cambodia. BMC Pregnancy Childbirth.

[15] Tadesse Nigussie Tola AHT (2015). Cultural malpractices during pregnancy, child birth and postnatal period among women of child bearing age in Limmu Genet Town, Southwest Ethiopia. Sci J Publ Health.

[16] MOH: The implementation of Ethiopia's Health Extension Program. Addis Ababa; 2016.

[17] Gedamu H, Tsegaw A, Debebe E (2018). The prevalence of traditional malpractice during pregnancy, child birth, and postnatal period among women of childbearing age in Meshenti Town, 2016. Int J Reprod Med.

[18] Ayaz S, Yaman Ş (2008). Potentially harmful traditional practices during pregnancy and postpartum. Eur J Contraception Reprod Health Care.

[19] Begashaw B, Tesfaye T (2016). Healthcare utilization among urban and rural households in Esera District: comparative cross-sectional study. Am J Public Health Res.

[20] Taddese Alemu Zerfu MUaKB: Dietary habits, food taboos, and perceptions towards weight gain during pregnancy in Arsi, rural central Ethiopia: a qualitative cross-sectional study. J Health Popul Nutr. 2016;35(22). 10.1186/s41043-016-0059-8 PMC5025964 27456151

[21] CSA: Population census. Ethiopia; 2007.

[22] Gabbe SG: Gabbe obstetrics, Normal labour and delivery, 6 edn. Philadelphia; 2012.

[23] al.] SGGe: Gabbe obstetrics, Normal labour and problem labour, 6th edn. Philadelphia; 2012.

[24] Sharma S, Van Teijlingen E, Hundley V, Angell C, Simkhada P: Dirty and 40 days in the wilderness: Eliciting childbirth and postnatal cultural practices and beliefs in Nepal. BMC Pregnancy Childbirth 2016;16. 10.1186/s12884-016-0938-4 PMC4933986 27381177

[25] Devkota B, Maskey J, Pandey AR, Karki D, Godwin P, Gartoulla P, Mehata S, Aryal KK (2020). Determinants of home delivery in Nepal – A disaggregated analysis of marginalised and non-marginalised women from the 2016 Nepal Demographic and Health Survey. Plos One.

[26] McNamara K, Wood E (2019). Food taboos, health beliefs, and gender: understanding household food choice and nutrition in rural Tajikistan. J Health Popul Nutr.

[27] ARCHIVE - Living Wage Series - Ethiopia - January 2018 - In Ethiopian Birr, per Month

[28] Chernet AG, Dumga KT, Cherie KT (2019). Home delivery practices and associated factors in Ethiopia. J Reprod Infertil.

[29] Ogolla JO (2015). Factors associated with home delivery in West Pokot County of Kenya. Adv Public Health.

[30] Gebregziabher NK, Zeray AY, Abtew YT, Kinfe TD, Abrha DT (2019). Factors determining choice of place of delivery: analytical cross-sectional study of mothers in Akordet town, Eritrea. BMC Public Health.

[31] O’zsoy SA, Katabi V (2008). A comparison of traditional practices used in pregnancy, labour and the postpartum period among women in Turkey and Iran. Midwifery.

[32] Mitiku AA, Dimore AL, Mogas SB (2020). Determinants of home delivery among mothers in Abobo District, Gambella Region, Ethiopia: a case control study. Int J Reprod Med.

[33] Mugo NS, Agho KE, Zwi AB, Dibley MJ (2016). Factors associated with different types of birth attendants for home deliveries: an analysis of the cross-sectional 2010 South Sudan household survey. Glob Health Action.

[34] Scott NA, Henry EG, Kaiser JL, Mataka K, Rockers PC, Fong RM, Ngoma T, Hamer DH, Munro-Kramer ML, Lori JR (2018). Factors affecting home delivery among women living in remote areas of rural Zambia: a cross-sectional, mixed-methods analysis. Int J Womens Health.

[35] Salehudin RAGaS: Traditional belief and practice on postpartum recovery among mothers in East Coast of Peninsular Malaysia. MUCET 2017.

[36] Nigussie T, Henok A (2015). Cultural malpractices during pregnancy, child birth and postnatal period among women of child bearing age in Limmu Genet Town, Southwest Ethiopia. Sci J Public Health.

[37] Getnet W, Aycheh W, Tessema T (2018). Determinants of food taboos in the pregnant women of the Awabel District, East Gojjam Zone, Amhara Regional State in Ethiopia. Adv Public Health.

[38] Tela FG, Gebremariam LW, Beyene SA (2020). Food taboos and related misperceptions during pregnancy in Mekelle city, Tigray, Northern Ethiopia. Plos One.

[39] Mesele HA. Traditional maternal health beliefs and practices in Southern Tigray. Anat Physiol. 2018;8(298).

[40] Lamxay V, de Boer HJ, Björk L (2011). Traditions and plant use during pregnancy, childbirth and postpartum recovery by the Kry ethnic group in Lao PDR. J Ethnobiol Ethnomed.

[41] Zenebe K, Alem H: Prevalence of Cultural Malpractice and Associated Factors among Women Attending MCH Clinic at Debretabor Governmental Health Institutions South Gondar, Amhara Region, North West Ethiopia, 2015. Gynecol Obstetr. 2016;2016(04).

[42] Kassahun G, Wakgari N, Abrham R (2019). Patterns and predictive factors of unhealthy practice among mothers during pregnancy, childbirth, postnatal and newborn care in Southern Ethiopia: a community based cross-sectional study. BMC Res Notes.

[43] Adokiye EA, Isioma AJ Levi WO: Influence of culturally-based abdominal massage and antenatal care uptake among pregnant women in a tertiary hospital in Southern Nigeria. Brit J Med Medical Res. 2016;18(6).

